# Quantitative structure–activity relationship modeling for predication of inhibition potencies of imatinib derivatives using SMILES attributes

**DOI:** 10.1038/s41598-022-26279-8

**Published:** 2022-12-15

**Authors:** Hamideh Hamzehali, Shahram Lotfi, Shahin Ahmadi, Parvin Kumar

**Affiliations:** 1 Department of Chemistry, East Tehran Branch, Islamic Azad University, Tehran, Iran; 2 Department of Chemistry, Payame Noor University (PNU), Tehran, 19395-4697 Iran; 3 Department of Pharmaceutical Chemistry, Faculty of Pharmaceutical Chemistry, Tehran Medical Sciences, Islamic Azad University, Tehran, Iran; 4 Department of Chemistry, Kurukshetra University, Kurukshetra, Haryana 136119 India

**Keywords:** Cancer therapy, Drug screening, Molecular medicine, Theoretical chemistry, Computational chemistry

## Abstract

Chronic myelogenous leukemia (CML) which is resulted from the BCR-ABL tyrosine kinase (TK) chimeric oncoprotein, is a malignant clonal disorder of hematopoietic stem cells. Imatinib is used as an inhibitor of BCR-ABL TK in the treatment of CML patients. The main object of the present manuscript is focused on constructing quantitative activity relationships (QSARs) models for the prediction of inhibition potencies of a large series of imatinib derivatives against BCR-ABL TK. Herren, the inbuilt Monte Carlo algorithm of CORAL software is employed to develop QSAR models. The SMILES notations of chemical structures are used to compute the descriptor of correlation weights (CWs). QSAR models are established using the balance of correlation method with the index of ideality of correlation (IIC). The data set of 306 molecules is randomly divided into three splits. In QSAR modeling, the numerical value of R^2^, Q^2^, and IIC for the validation set of splits 1 to 3 are in the range of 0.7180–0.7755, 0.6891–0.7561, and 0.4431–0.8611 respectively. The numerical result of ${CR}_{p}^{2}$ > 0.5 for all three constructed models in the Y-randomization test validate the reliability of established models. The promoters of increase/decrease for pIC_50_ are recognized and used for the mechanistic interpretation of structural attributes.

## Introduction

BCR-ABL tyrosine kinase (TK) oncoprotein as an oncogene is present in 95% of patients suffering from chronic myeloid leukemia (CML). Therefore, tyrosine kinase inhibitors (TKIs), such as imatinib as the first drug against the BCR-ABL TK, have been used in the therapy of most cases of CML patients. Imatinib competitively targets the ATP-binding site in the TK domain of the BCR-ABL oncoprotein and reduces the activity of BCR-ABL. Due to the point mutations in the BCR-ABL kinase domain, some patients particularly in the advanced phases of CML, develop imatinib resistance. Therefore, to overcome imatinib resistance, novel analogues of Imatinib such as ponatinib, nilotinib, dasatinib, bosutinib, etc., have been developed as TKIs and tested in patients with BCR-ABL positive CML. Hence, the development and design of more potent BCR-ABL TKIs, specifically imatinib derivatives is a matter of great importance and would help in the therapeutic treatments of CML patients^1–5^.

Quantitative structure–activity relationship (QSAR) is an approach that can be applied to the construction of pharmacophore models, new drug discovery, and assessment of the activity/behavior of compounds^6–8^. Also, QSAR is a predictive and diagnostic process employed for finding quantitative relationships between chemical structures and biological activity or property. QSAR is the concluding outcome of computational methods that begin with an appropriate molecular structure description and conclude with some interpretation, assumption, and judgments on the behaviour of molecules in the biological and physicochemical under examination^9,10^. Finding a class of molecular descriptors that indicates variations in the structural properties of the molecule, is the main goal of QSAR model development.

The Monte Carlo algorithm of CORrelation And Logic (CORAL) software has been applied for QSAR modeling of different endpoints^11–15^. Random distribution of dataset into training and validation subsets, production of optimal descriptors of correlation weights (DCW), and the construction of predictive models using the physicochemical conditions of corresponding experiments are unique options available in the CORAL software for the development of QSAR models^16–22^. The literature survey shows that the Index of Ideality of Correlation (IIC) has been applied to improve the statistical result of the QSAR model^23–28^. In addition, the most descriptors used in common QSAR models do not have physical meaning and can not be associated with mechanistic interpretation. It has to be noted that QSAR models developed with CORAL software are developed with SMILES notation based molecular descriptors that have mechanistic interpretation and could be associated with molecular fragments.

The objective of the present work is to apply the inbuilt Monte Carlo algorithm of CORAL software for the building QSAR model to predict inhibition potencies (pIC_50_) of 306 Imatinib derivatives against BCR-ABL tyrosine kinase (TK). The balance of correlation method with IIC is used to develop QSAR models. The reliability and predictability of the designed QSAR model are assessed by three random splits.

## Method

### Data

Zin et al.^29^ had extracted the inhibition potential of 306 compounds for the human BCR-ABL tyrosine-kinase from the ChEMBL v23 (2017) database^30^. The inhibition potential of compounds was defined as half maximal inhibitory concentration in mol/L (IC_50_). Additionally, the inhibition experimental data of BCR-ABL tyrosine kinase was transformed to a negative logarithm value (pIC_50_ ). The endpoint pIC_50_ was taken as the dependent parameter for constructing QSAR models. The range of pIC_50_ was between 9.37 and 4.03. Three splits were created form the dataset (n = 306) and the compounds of each split was randomly divided into the training (34%), invisible training (35%), calibration (15%) and validation (16%) sets. The SMILES notations, split distribution, experimental pIC_50_, predicted pIC_50,_ and applicability domain of each compound are depicted in Table S1. The task of each set in developing the QSAR models was already described in the literature^31,32^.

### Optimal SMILES-based descriptors

In the CORAL software, three types of optimal descriptors i.e. SMILES-based, graph-based and hybrid descriptors (combination of SMILES and Graph) can be employed to develop QSAR models.

The optimal descriptor is a mathematical function of so-called correlation weights (CW). Correlation weights are numerical coefficients associated with various molecular features extracted from SMILES symols. In other words, the univariate models investigated in this research are based on the “descriptors of correlation weights” (DCW). The Monte Carlo algorithm was used to calculate the DCW. In the present research, the SMILES-based descriptor was employed to make the QSAR models. The optimal descriptors used to build pIC_50_ models are calculated as follows:

1 $$\mathrm{DCW}\left({\mathrm{T}}^{*}, {\mathrm{N}}^{*}\right)={}^{\mathrm{SMILES}}\mathrm{DCW}\left({\mathrm{T}}^{*}, {\mathrm{N}}^{*}\right)$$

2 $${}^{\mathrm{SMILES}}\mathrm{DCW}{}_{ }{}^{ }({\mathrm{T}}^{*}, {\mathrm{N}}^{*})=\sum \mathrm{CW}\left({\mathrm{SSS}}_{\mathrm{K}}\right)+\mathrm{CW}\left(\mathrm{HALO}\right)+\mathrm{CW}\left(\mathrm{NOSP}\right)+\mathrm{CW}\left(\mathrm{HARD}\right)+\mathrm{CW}\left(\mathrm{PAIR}\right)+\mathrm{CW}\left({\mathrm{C}}_{\mathrm{max}}\right)+\mathrm{CW}\left({\mathrm{N}}_{\mathrm{max}}\right)+\mathrm{CW}\left({\mathrm{O}}_{\mathrm{max}}\right)$$

Here, T is the notation of threshold and N is the notation of the number of epochs. The T is an integer utilized to split SMILES attributes (i.e. Sk, SSk, and SSSk) into two classes i.e. active and rare. If a molecular attribute, A, takes place less than T times, then this molecular attribute should be omitted from the construction of the model ( molecular attribute is calculated from SMILES in the training set), hence the correlation weight of the A, CW(A) = 0. Therefore, this molecular attribute has been distinguished as rare. The T* and N* are the numerical values of the T and N that yield the best statistical result of a model for the calibration set.

The details of notation given in Eq. (2) are as follows: SSS_k_, a local SMILES attribute, is a combination of three SMILES atoms; NOSP, HALO, and BOND are global SMILES attributes that display the existence or absence of nitrogen (N), oxygen (O), sulfur (S), and phosphorus (P) (NOSP), fluorine, chlorine, and bromine (HALO); BOND illustrates the presence or absence of double (‘ = ’), triple (‘#’) and stereochemical (‘@’ or ‘@@)’ bonds; PAIR imply the combination of BOND and NOSP; HARD displays the presence or existence of NOSP, HALO, and BOND; C_max_ represents the maximum number of rings; N_max_ and O_max_ are the total numbers of nitrogen and oxygen atoms in the molecular structure. The CW(A) demonstrates the correlation weight for the SMILES-attributes e.g. SSS_k_, NOSP, BOND, HALO, PAIR, Cmax, Nmax, and Omax. These correlation weights are calculated using the Monte Carlo optimization^33–37^.

The obtained numerical data in terms of DCW is used to determine the inhibition potential for Imatinib derivatives (pIC_50_) by the least square method using the following one-variable model:

3 $${pIC}_{50}={\mathrm{C}}_{0}+{\mathrm{C}}_{1}\times \mathrm{DCW}\left({\mathrm{T}}^{*}, {\mathrm{N}}^{*}\right)$$

### Monte Carlo optimization

In the present research modified target function (TF_m_) i.e. the balance of correlation with IIC was employed to compute the DCW^32^. The following mathematical relationships are used to compute TF_m_:

4 $$TF={R}_{training}+{R}_{invTraining}-\left|{R}_{training}-{R}_{invTraining}\right|\times Const$$

5 $${TF}_{m}=TF+{IIC}_{CAL} \times Const$$

Here, *R*_*training*_ and *R*_*invTraining*_ indicate the correlation coefficients for the training and invisible training sets, respectively. The empirical constant (Const) is usually fixed.

The index of ideality if correlation for the calibration set (IIC_CAL_) is calculated using the following equation:

6 $$\mathrm{IIC}={\mathrm{R}}_{\mathrm{C}AL}\times \frac{\mathrm{min}({}^{-}{\mathrm{MAE}}_{\mathrm{CAL}}, {}^{+}{\mathrm{MAE}}_{\mathrm{CAL}})}{\mathrm{max}({}^{-}{\mathrm{MAE}}_{\mathrm{CAL}}, {}^{+}{\mathrm{MAE}}_{\mathrm{CAL}})}$$

7 $${}^{-}{\mathrm{MAE}}_{\mathrm{CLB}}=-\frac{1}{\mathrm{N}}\sum_{y=1}^{{N}^{-}} \left|{\Delta }_{\mathrm{k}}\right| \quad {\Delta }_{\mathrm{k}}<0, {}^{-}\mathrm{N \,  is \, the \, number \, of } \, {\Delta }_{\mathrm{k}}<0$$

8 $${}^{+}{\mathrm{MAE}}_{\mathrm{CLB}}=+\frac{1}{\mathrm{N}}\sum_{y=1}^{{N}^{+}}\left|{\Delta }_{\mathrm{k}}\right| \quad {\Delta }_{\mathrm{k}}\ge 0, {}^{+}\mathrm{N \, is \, the \, number \, of } \, {\Delta }_{\mathrm{k}}\ge 0$$

9 $${\Delta }_{\mathrm{k}}={\mathrm{Observed}}_{\mathrm{k}}-{\mathrm{Calculated}}_{\mathrm{k}}$$

The ‘k’ is the index (1, 2, …. N). The observed_k_ and calculated_k_ are related to the endpoint.

### Applicability domain

According to the 3rd principle of the OECD, the applicability domain (AD) is recommended for the validation of the established QSAR model. The physicochemical, structural, or biological space, knowledge, or information on which the model's training set was created and for which it is used to generate predictions about new compounds is known as the AD^38,39^.

In the CORAL program, Monte Carlo-based QSAR, scattering of SMILES attributes in the training, invisible training and calibration sets is utilized to achieve AD^40,41^. If a substance does not fall within the scope of AD, it is identified as an outlier and cannot be associated with a reliable prediction.

In CORAL, a compound is recognized in the scope of AD if the following inequality is fulfilled, otherwise, it is recognized as an outlier:

10 $${\mathrm{Defect}}_{\mathrm{molecule}} <2\times {\overline{\mathrm{Defect}} }_{TRN}$$

where ${\overline{\mathrm{Defect}} }_{\mathrm{TRN}}$ is an average of the statistical defect (D) for the dataset of the training set.

The statistical defect (D) can be described as the sum of statistical defects of all attributes present in the SMILES notation.

11 $${\mathrm{Defect}}_{\mathrm{Molecule}}=\sum_{\mathrm{k}=1}^{N{\mathrm{A}}}{\mathrm{Defect}}_{{\mathrm{A}}_{\mathrm{K}}}$$

NA is the number of active SMILES attributes for the given compounds.

The “*statistical defect,*” *Defect(A)* for an attribute of SMILES can be defined by the following mathematical equation:

12 $${\mathrm{Defect}}_{{\mathrm{A}}_{\mathrm{K}}}=\frac{\left|{\mathrm{P}}_{\mathrm{TRN}}{(\mathrm{A}}_{\mathrm{K}})-{\mathrm{P}}_{\mathrm{CAL}}{(\mathrm{A}}_{\mathrm{K}})\right|}{{\mathrm{N}}_{\mathrm{TRN}}{(\mathrm{A}}_{\mathrm{K}})+{\mathrm{N}}_{\mathrm{CAL}}{(\mathrm{A}}_{\mathrm{K}})} \quad \mathrm{ If }{\mathrm{A}}_{\mathrm{K}}>0$$

$${\mathrm{Defect}}_{{\mathrm{A}}_{\mathrm{K}}}=1 \quad \mathrm{ If }{\mathrm{A}}_{\mathrm{K}}=0$$

${P}_{TRN}{(A}_{K})$ and ${P}_{TCAL}{(A}_{K})$ are the probability of an attribute 'A_k_' in the training and the calibration sets; ${N}_{TRN}{(A}_{K})$ and ${N}_{CAL}{(A}_{K})$ are the number of times of A_k_ in the training and calibration sets, respectively.

### Validation of the model

The statistical eminence of the created QSAR models for pIC_50_ of Imatinib derivatives is evaluated on the basis of the three methodologies: (i) internal validation or cross-validation by determining the R^2^, IIC, CCC, Q^2^, and F-test on the training set; (ii) external validation by determining the Q^2^F_1_, Q^2^F_2_, Q^2^F_3_, CRp^2^, s, MAE, r̅_m_^2^, and Δr_m_^2^ utilizing the test set substances and (iii) data randomization or Y-scrambling (Table 1). The mathematical relationship of these statistical parameters has been provided in the literature^42–46^. In Table 1, Y_obs_ is observation endpoint; Y_prd_ is the prediction endpoint; R^2^ and ${R}_{0}^{2}$ are the squared correlation coefficient values between the observed and predicted endpoints with intercept and without intercept respectively, and ${R}_{r}^{2}$ is squared mean correlation coefficient of randomized models.

**Table 1:** The mathematical equation of different statistical benchmark of the predictive potential for CORAL models.

Type of validation	Criterion of the predictive potential
Internal	${R}^{2}=1-\frac{\sum {({Y}_{obs}-{Y}_{prd})}^{2}}{\sum {({Y}_{obs}-\overline{Y })}^{2}}$
	$${Q}^{2}=1-\frac{\sum {({Y}_{prd}-{Y}_{obs})}^{2}}{\sum {({Y}_{obs}-{\overline{Y}}_{train})}^{2}}$$
External	${Q}_{F1}^{2}=1-\frac{\sum {({Y}_{per\left(test\right)}-{Y}_{obs\left(test\right)})}^{2}}{\sum {({Y}_{obs\left(test\right)}-{\overline{Y}}_{train})}^{2}}$
	$${Q}_{F2}^{2}=1-\frac{\sum {({Y}_{prd\left(test\right)}-{Y}_{obs\left(test\right)})}^{2}}{\sum {({Y}_{obs\left(test\right)}-{\overline{Y}}_{ext})}^{2}}$$
	$${Q}_{F3}^{2}=1-\frac{\sum {({Y}_{prd\left(test\right)}-{Y}_{obs\left(test\right)})}^{2}/{n}_{ext}}{\sum {({Y}_{obs\left(test\right)}-{\overline{Y}}_{train})}^{2}/{n}_{train}}$$
	$${R}_{m}^{2}={R}^{2}\times \left(1-\sqrt{{R}^{2}-{R}_{0}^{2}}\right)$$
	$$CCC=\frac{2\sum (X-\overline{X })(Y-\stackrel{-}{Y)}}{\sum {(X-\overline{X })}^{2}+\sum {(Y-\overline{Y })}^{2}+n({(\overline{X }-\overline{Y })}^{2}}$$
	$$MAE=\frac{1}{n}\times \sum \left|{Y}_{obs}-{Y}_{prd}\right|$$
Y-randomization	${C}_{{R}_{p}^{2}}=R\sqrt{\left({R}^{2}-{R}_{r}^{2}\right)}$

## Results and discussion

### QSAR models

With the mentioned data in “Data”, three splits were generated randomly. Each split was further divided into four sets namely training, invisible training, calibration and validation sets. To establish the QSAR model, a balance of correlation with the IIC technique was employed. The values of IIC_weight_ (weight of IIC) and dR_weight_ (weight for dR in the balance of correlations) were 0.2, and 0.1, respectively. The result for the preferable T* and N* was 1 and 15 for all splits. With the best-preferred values of T* and N*, the pIC_50_ (endpoint) for each split was computed and the developed QSAR models are as the following:

13 $$\mathrm{Split }1\quad  {pIC}_{50}=3.6679\left(\pm 0.0196\right)+0.2889(\pm 0.0016)\times DCW(1, 15)$$

14 $$\mathrm{Split }2\quad  {pIC}_{50 }=1.5438\left(\pm 0.0259\right)+0.2660(\pm 0.0017)\times DCW(1, 15)$$

15 $$\mathrm{Split }3\quad  {pIC}_{50 }=3.4165\left(\pm 0.0126\right)+0.2696(\pm 0.0010)\times DCW(1, 15)$$

The statistical characteristics of the generated QSAR models computed by relationships 13–15 are depicted in Table 2. The outcomes in Table 2 demonstrate that all generated QSAR models from the statistical point of view are appropriate and match the requirements of various validation criteria. The robustness of established QSAR models was demonstrated by the numerical value of R^2^ and Q^2^ values which were more than 0.5 and 0.7^47,48^. In addition, the numerical value of the R^2^m metric for the validation set of all designed QSAR models was satisfactory and follows the criteria suggested by Roy et al.^49^. Also, the ${\overline{R} }_{m}^{2}$-scaled and ${\Delta R}_{m}^{2}$-scaled introduced as modified R^2^m metric by Roy et al. were computed^50^, these values were 0.6928 and 0.0216, 0.6878 and 0.0929, and 0.7339 and 0.1230 for split 1 to 3, respectively. The trustworthiness of the constructed QSAR models was also confirmed by the Y-randomization test.

**Table 2:** The summary statistical characteristics and criteria of predictability of the QSAR models for three random splits.

Split	Set	n	R^2^	CCC	IIC	Q^2^	${Q}_{F1}^{2}$	${Q}_{F2}^{2}$	${Q}_{F3}^{2}$	${R}_{m}^{2}$	${CR}_{p}^{2}$	${\overline{R} }_{m}^{2}$	${\Delta R}_{m}^{2}$	S	MAE	F
1	Training	105	0.7785	0.8755	0.8021	0.7691					0.7757			0.630	0.512	362
	Invisible training	107	0.7783	0.8533	0.6423	0.7703					0.7745			0.661	0.536	369
	Calibration	47	0.8473	0.9130	0.9205	0.8343	0.8507	0.8448	0.8601	0.6664	0.8398	0.7394	0.1461	0.503	0.413	250
	Validation	47	0.7755	0.8762	0.8611	0.7561				0.6499		0.6835	0.0672	0.5634	0.4587	-
2	Training	94	0.8353	0.9102	0.7382	0.8282					0.8328			0.574	0.446	466
	Invisible training	98	0.7882	0.8837	0.7953	0.7799					0.7661			0.565	0.436	357
	Calibration	56	0.8070	0.8934	0.8982	0.7953	0.8077	0.8057	0.8061	0.7961	0.7703	0.7230	0.1462	0.578	0.432	226
	Validation	57	0.7180	0.8463	0.4708	0.6891				0.5761		0.6095	0.0669	0.7092	0.5371	-
3	Training	104	0.8058	0.8924	0.7997	0.7996					0.8796			0.619	0.487	423
	Invisible training	95	0.8060	0.8641	0.5237	0.7980					0.8742			0.627	0.480	386
	Calibration	61	0.7579	0.8696	0.8699	0.7403	0.7427	0.7417	0.7968	0.6589	0.8049	0.6612	0.0047	0.613	0.485	185
	Validation	46	0.7680	0.8202	0.4431	0.7468				0.7473		0.6437	0.2072	0.6972	0.5274	-

After several repetitions of new random models were developed and the values of R^2^ were found below 0.1 (see Table S2 as supplementary information). These result indicates that the correlation between pIC_50_ and molecular attributes is not based on chance correlation. Moreover, for three splits, the CR^2^p was obtained greater than 0.75, which confirmed the non-chance correlation of developed models^51^.

The AD for each compound in models 1 to 3 shown in Table S1 based on the results of defectvalue. The percentages of compounds in the AD of models were 81, 83, and 87% for splits 1–3, respectively. It showed that the three prediction models were able to predict more than 80% of the new data.

**Figure 1:**
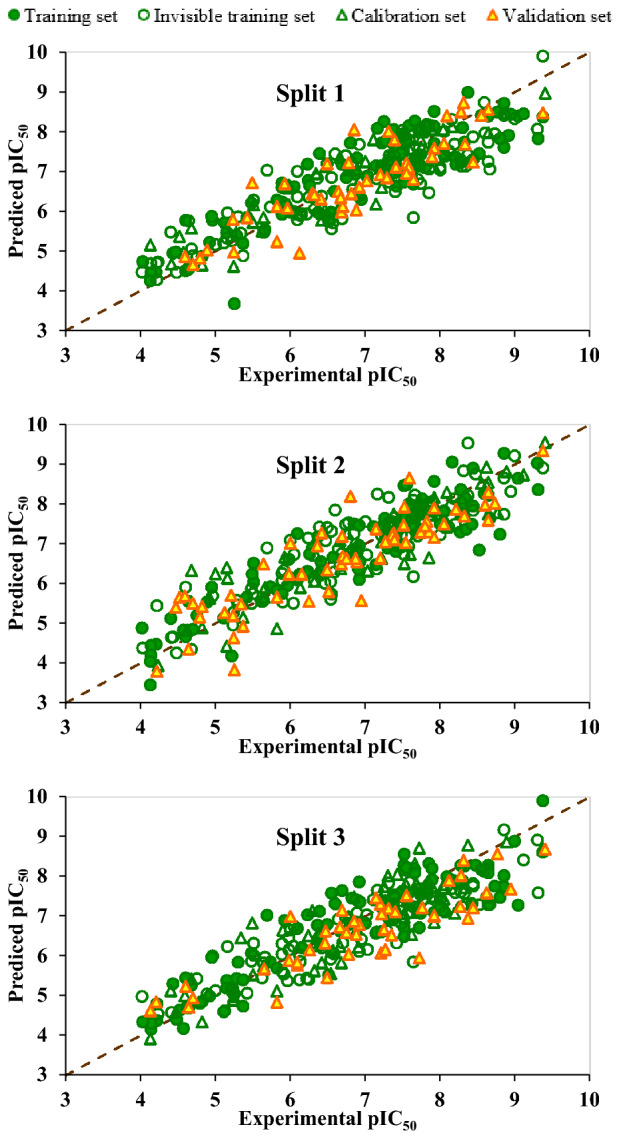
The graph of the experimental versus predicted values of pIC_50_ for split 1 to split 3.

**Figure 2:**
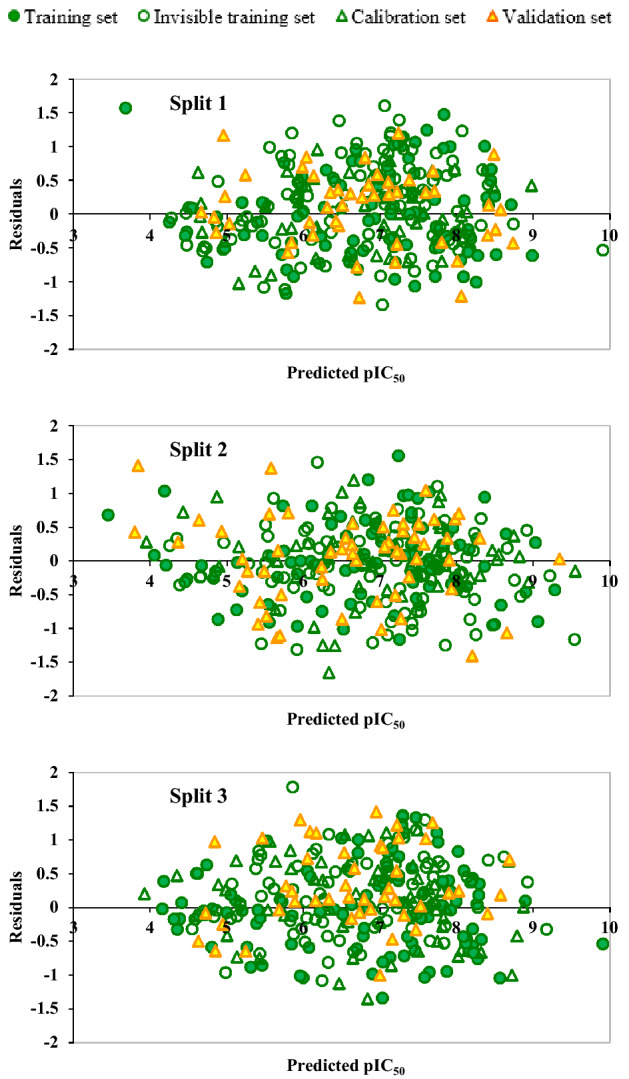
The graph of the residuals versus predicted values of pIC50 for split 1 to split 3.

Figures 1 and 2 demonstrate the pictorial presentation of experimental data of pIC50 versus predicted pIC50 and residual pIC50 versus predicted pIC50 of three models. As can be seen in Fig. 1, there is good agreement between experimental and predicted data in the suggested models. It can also be seen in Fig. 2 that the dispersion of residual pIC50 near the horizontal line centred around zero. All these results confirmed that all constructed QSAR models were robust and well fitted.

### Interpretation of the QSAR model

Mechanistic interpretation of models helps in understanding the effectiveness of descriptors in the predicted endpoint. The mechanistic interpretation of built-up QSAR models utilizing the CORAL program is done with correlation weights (CW) of SMILES-attributes which are achieved from several runs of the Monte Carlo optimization. The CW for each SMILES attributes in various probs of a model likely positive, negative, or both positive and negative. The positive and negative promoters are considered as promoters of increase and decrease of the activity or an endpoint, respectively. Consequently, promoters of increase of pIC50 have positive CW and promoters of decrease of pIC_50_ have negative CW. But, if the structural attribute in all runs both positive and negative values of CW, then these attributes are undefined. Table 3 represents the list of the structural features as the promoters of increase or decrease of pIC_50_ achieved in the results of three probs of the Monte Carlo optimization with optimum T* and N* along with the interpretation of the promoters (NT is number of attributes in the training set, NiT is number of attributes in the invisible training set, and NC is number of attributes in the calibration set). According to the results, the important SMILES-descriptors as the promoter of increase/decrease of pIC_50_ were distinguished and recognized. The SMILES-based descriptors as promoters of increase of pIC_50_ were c…c…c…, c…c…1… and Cmax.3……, and the promoter of decrease pIC_50_ was C…(…(….

**Table 3:** List of structural attributes (SAk) as a promoter of increase/decrease extracted from three split of the constructed model.

No	Structural attributes (SAk)	Split	CWs Probe 1	CWs Probe 2	CWs Probe 3	NT	NiT	NC	Defect [SAk]	Comments
**Promoter of increase**										
1	c…c…c…	1	0.29321	0.31987	0.17186	103	103	43	0.0005	Three successive aromatic carbon
		2	0.13281	0.15897	0.13236	92	95	52	0.0003	
		3	0.41397	0.00538	0.44783	100	92	60	0.0001	
2	c…c…1…	1	0.491	0.18688	0.28507	80	82	35	0.0001	Two successive aromatic carbon in ring no. 1
		2	0.28522	0.33232	0.08556	74	70	45	0.0001	
		3	0.07861	0.25241	0.07268	47	46	30	0.0005	
3	Cmax.3……	1	1.36827	0.17551	0.3596	54	56	25	0.0002	Maximum no. of cycles in compound
		2	0.05655	1.10274	0.62154	52	49	27	0.0009	
		3	1.17714	2.22122	0.88535	52	42	32	0.0003	
**Promoter of decrease**										
1	C…(…(…	1	−0.0558	−0.3626	−0.06209	7	9	3	0.0003	Aliphatic carbon with two branching
		2	−0.16545	−0.21343	−0.12731	8	4	4	0.0011	
		3	−0.06639	−0.30183	−0.2403	6	6	6	0.0034	

### Comparison with prior reports

Kyaw Zin and colleagues^29^ reported a QSAR model by the same data relying on deep neural nets (DNN) and hybrid sets of 2D/3D/MD descriptors to predict the inhibition potencies of 306 imatinib derivatives. The dataset was divided into two sets i.e. training set (260 compounds) and a test set (46 compounds). They built multiple DNN and RF regressors with hybrid 2D/3D/MD descriptors and showed high predictive power through rigorous validation tests. Through rigorous validation tests, they reported that their DNN regression models resulted excellent external prediction performances for the pIC_50_ data set. The R^2^ of training and validation setes was 0.99 and 0.68 respectively and the MAE of training and test set was 0.08 and 0.67 respectively.

The comparison QSAR model here with the previous study showed that the structure, physicochemical parameters or previous calculations of the chemicals descriptors for the construction of the models were required by the model, while in the case of CORAL software, a text file containing SMILES notations of compounds and endpoint was used for model development. Here, we used 3 splits to establish three QSAR models using four sets (training, invisible training, calibration and validation set), but in previously constructed models, a single split utilizing two sets (training and test set) was used. In the present research, the molecular features responsible for the increase/decrease of endpoint were also detected for mechanistic interpretation.

In terms of statistical characterization, the proposed QSAR model by CORAL for the prediction of pIC_50_ was superior to the reported model. The statistical parameters ${Q}_{F1}^{2}$, ${Q}_{F2}^{2}$, ${Q}_{F3}^{2}$, ${CR}_{p}^{2}$, CCC and IIC were not reported in the previous report. The R^2^ of training and validation setes for split 1 to 3 are between 0.76–0.85 and 0.71–0.78, respectively and the MAE of training and validation sets for split 1 to 3 are between 0.41–0.54 and 0.46–0.54, respectively. Therfore, the QSAR models established here are more reliable and have better predictability.

## Conclusion

In this work, to predict pIC_50_ of 306 Imatinib derivatives, QSAR models were created using the Monte Carlo method and validated with several parameters. The QSAR models were established using a modified target function (TF_m_). The statistical characterization of constructed models was justified using internal and external validation metrics such as R^2^, IIC, CCC, Q^2^, ${Q}_{F1}^{2}$, ${Q}_{F2}^{2}$, ${Q}_{F3}^{2}$, F, s, MAE, RMSE, $\overline{{R }_{m}^{2}}$, $\overline{{\Delta R }_{m}^{2}}$, scaled-$\overline{{\mathrm{R} }_{\mathrm{m}}^{2}}$, scaled-$\overline{\Delta {\mathrm{R} }_{\mathrm{m}}^{2}}$, ${CR}_{p}^{2}$, and Y-randomization test. In the constructed QSAR model, the numerical value of R^2^, Q^2^, and IIC for the validation set of splits 1 to 3 were in the range of 0.7180- 0.7755, 0.6891–0.7561, and 0.4431–0.8611 respectively. The domain of applicability (AD) was applied to identify the outliers in the generated QSAR models. The structural features as promoters of pIC_50_ increase/decrease were also identified.

## Supplementary Information

Supplementary Table S1.

Supplementary Table S2.

## Data Availability

The datasets used and/or analyzed during the current study are available from the corresponding author on reasonable request.
